# Personality, depression and anxiety in primary Sjogren's syndrome – Association with sociodemographic factors and comorbidity

**DOI:** 10.1371/journal.pone.0210466

**Published:** 2019-01-17

**Authors:** Vera Milic, Milica Grujic, Jasmina Barisic, Jelena Marinkovic-Eric, Dragana Duisin, Andja Cirkovic, Nemanja Damjanov

**Affiliations:** 1 Department of Rheumatology, Faculty of Medicine, University of Belgrade, Belgrade, Serbia; 2 Department of Psychiatry, Clinical Centre of Serbia, Belgrade, Serbia; 3 Department of Medical Statistics, Faculty of Medicine, University of Belgrade, Belgrade, Serbia; University of Florence, ITALY

## Abstract

**Objective:**

Patients with primary Sjögren’s Syndrome (pSS) have diminished health quality and fatigue, arthralgia along with dryness of the mouth and eyes have major impact on their psychological and social aspects of life. The purpose of this study was to determine psychological features of patients with pSS. We analyzed personality, depression and anxiety of patients with primary Sjögren’s Syndrome (pSS) in comparison with patients with rheumatoid arthritis (RA) and healthy controls (HC) and assessed their association with sociodemographic factors and comorbidity.

**Methods:**

In 105 pSS patients (mean age 51.34 years, mean disease duration 5.98 years), 52 RA patients (mean age 51.37 years, mean disease duration 8.10 years) and 54 HC (mean age 51.35 years) clinical and sociodemographic characteristics were determined and results analyzed. At enrollment patients and controls completed the Revisited NEO Personality Inventory Five-Factor model (NEO-PI-R), the Zung Self-Rating Depression Scale and the Zung Self-Rating Anxiety Scale. Statistical analyses were performed using SPSS [Version 16.0]. The relative size of the effect was assessed based on standardized estimates of effect size (d).

**Results:**

Patients with pSS, similarly to RA patients had higher scores of Neuroticism (d = 0.46, p = 0.007) and lower scores of Extraversion (d = 0.51, p = 0.001) and Openness for experience (d = 0.65, p = 0.013) compared to HC. There was no significant differences between pSS group and HC in the depression (d = 0.171, p>0.05). However, patients with pSS had higher anxiety in comparison to HC (p<0.0001). In multivariate models, education and satisfaction with family relationships were significant predictors for psychological characteristics of patients, independently of clinical diagnosis.

**Conclusions:**

Our study is the first to show that patients with pSS scored high on neuroticism and anxiety and low on sociability. Education and satisfaction with family relationships predisposed to their psychological profile. Psychological assessment of patients with pSS may improve understanding and treatment of this clinical condition.

## Introduction

Primary Sjogren syndrome (pSS) is an autoimmune chronic inflammatory disease characterized by focal lymphocytic infiltration of exocrine glands and other tissues. The main symptoms of pSS are dryness of the mouth and eyes, arthralgiae and fatigue. These symptoms are present in almost all patients, while severe systemic manifestations occur in approximately 25% of patients with pSS [1]. Patients with pSS have reduced quality of life and typical complaints of fatigue and arthralgia along with dryness of the mouth, eyes, skin, vagina and airways significantly affect physical, psychological and social aspects of life [2]. The reported data suggest that patients with pSS have higher degree of distress and a lower sense of well-being than patients with rheumatoid arthritis (RA) [3].

Mood, sleeping and several neuropsychologic domains such as cognition are affected by pSS. Specifically, difficulties with attention, focusing, memory and new learning are commonly reported problems [4,5]. Moreover, the personality traits of pSS patients can potentially interact with the subjective dryness symptoms and treatment outcome. Psychological factors may influence the ability of patients to cope with fatigue and its consequences [6] including negative cognitions such as catastrophizing [7], avoidance of psychical activity [8] and lack of social support or overprotection [9]. Karageorgas et al. [10] have shown that neuroticism, depression and fibromyalgia are independent contributors of SS-related fatigue.

Whether somatization reflects pre-existing personality disorder or influence development of chronic illnesses such as pSS is still a matter of debate. Numerous studies have highlighted the role of psychological factors and unsatisfactory adaptive coping strategies in the pathogenesis of chronic diseases [11]. Relationships between personality and chronic disorders are very complex and bidirectional. The personality is one of the primary factors resposible for human adaptation, interaction and behavior in different environments. However chronic disorders affect patients`s emotional status and has negative impact on their mood [12]. Personality traits can predict the onset of psychiatric symptomatology. Patients with pSS have an increased risk of newly diagnosed depressive and/or anxiety disorders and sleep disorders that may impair their quality of life [4,13,14]. Moreover, unpredictable course of the disease and increased risk of developing lymphoma may additionally affect psychological status of pSS patients [15]. Thus, analysis of personality traits of patients with chronic illness is needed and may be useful in clinical practice. Personality characteristics of pSS patients and their relative contribution to subjective symptoms of pSS have not been studied extensively. The importance of psychological factors in pSS patients is supported by two recent studies. Karaiskos et al. [16] described the neuroticism, psychoticism and obsessiveness as psychological traits in pSS patients, whereas van Leeuwen *et al*. [17] demonstrated functional, alexithymic, self reliant and disfunctional psychological profiles in pSS patients.

There are several approaches to personality description and measurement. One of the most commonly used method is Five-Factor model (FFM) of personality, also known as the Big Five [18]. The Big Five/FFM is used for the assessment of variability in individuals’ personalities using several of trait dimensions. In contrast to the numerous descriptive personality models, in this model five domains capture the most important, basic individual differences in personality traits and convincing empirical data show the stability of FFM including the data from studies involving subjects with different age, education level, socioeconomic status, mental health or cultural backgrounds.

The model identifies five broad personality domains that are assumed to have a biological origin and have remarkable stability across cultures and, in the same individuals, for up to 45-year intervals [19]. The five domains defined by FFM are Neuroticism (N), Extraversion (E), Openness to experience (O), Agreeableness (A) and Conscientiousness (C). These five domains are believed to represent potentially affective, behavioral and cognitive characteristics of adult personality. Several studies have demonstrated that the Big Five personality domains are relatively stable even in depression and personality disorders [20,21]. However, others have argued that personality is influenced by a depressive mood [22,23]. As personality traits are genetically determined and minimally changed by environmental factors across the life span we hypothesized that subjects with particular personality characteristics are more prone to the development of chronic autoimmune diseases. Increasing number of diseases is considered to have psychosomatic origin so that contemporary holistic treatment modalities that involve change of life attitudes may be applied.

The purpose of this study was to determine psychological features of patients with pSS. We analyzed personality, depression and anxiety of patients with primary Sjögren’s syndrome in comparison with patients with rheumatoid arthritis and healthy controls and assessed their association with sociodemographic factors and comorbidity.

## Patients and methods

The total sample (N = 211) consisted of 105 pSS patients, 52 RA patients and 54 healthy controls. All participants were females. The patients with pSS (mean age 51.34 years, mean disease duration 5.98 years, range 1–25 years) and RA patients (mean age 51.37 years, mean disease duration 8.10 years, range 1–25 years) were recruited from the Outpatient Clinic of the Institute of Rheumatology, School of Medicine, University of Belgrade, Serbia. Patients with pSS fulfilled the American-European Consensus Group criteria for pSS [24] and RA patients fulfilled the 1987 revised American College of Rheumatology criteria for RA [25]. The fifty-four healthy controls (HCs) (mean age 51.35 years) age and gender matched with pSS patients, were randomly selected among friends and relatives of the Institute of Rheumatology personnel. Exclusion criteria for participants were: psychotic symptoms, substance abuse and personality disorders. The presence of chronic pain of any etiology in healthy control group was considered an exclusion criteria. None of the subjects included in the study had a history of psychiatric disease (including major depression), addiction disorders or personality disorders. Written informed consent was obtained from all study participants before examination.

The study was conducted in accordance with the Declaration of Helsinki and approved by the Ethical Committees of the Institute of Rheumatology, Belgrade, Serbia (number 29/1-14).

## Materials and methods

Clinical and laboratory data of the study population, including serological tests for pSS patients were collected. The Schirmer’s test was performed in order to assess lacrimal dysfunction in all pSS patients. The results of 99mTc-pertechnetate salivary gland scintigraphy were available in 87/105 (82.6%) pSS patients. A minor salivary gland biopsy was performed in 83/105 (79.04%) pSS patients. In patients with pSS, the disease activity was measured by EULAR (European League Against Rheumatism) SS disease activity index (ESSDAI, max range 0–123) and EULAR SS Patient Reported Index (ESSPRI, max range 0–10) [26]. In RA patients, the disease activity was measured by DAS (Disease Activity Score) 28 [27]. During the clinical visits, the participants fulfilled four questionnaires. The first questionnaire addressed the main sociodemographic characteristics of the participants (educational level, occupational and marital status, residence, social support and comorbidity). The second questionnaire was the Revisited NEO Personality Inventory Five-Factor model (NEO-PI-R) [28] for the assessment of personality characteristics. The third and fourth questionnaires were Zung Self-Rating Depression Scale, (SDS) [29] and the Zung Self-Rating Anxiety Scale, (SAS) [30] for the assessment of depression and anxiety symptoms, respectively.

The Revisited NEO Personality Inventory [18] is the 240-item questionnaire developed through rational and factor analytic methods to measure the five major domains of personality: I) Neuroticism—tendency toward negative emotions (anxiety, hostility, depression) with high reactivity to physiological changes, emotional instability, vulnerability to stress, and an inclination toward impulsive behaviors. II) Extraversion—the attitude to experience positive emotions, warmth, excitement seeking, and activity. III) Openness to experience—describes tendencies toward imagination and fantasy, aesthetics, creativity, ideas and values, and thought flexibility. IV) Agreeableness—involves a pro-social, altruistic orientation towards others, trust, straightforwardness, and tender-mindedness; V) Conscientiousness—includes competence, order, self-discipline, and achievement striving. Each of the factors is represented by six facet subscales that provide a more 'fine-grained' analysis of specific traits than the higher-order factors. Items on the measure are answered on a 5-point Likert scale ranging from 'strongly agree' to 'strongly disagree'. Each domain is classified into categories. For Neuroticism, average and low category was score <96 while high and very high was ≥96. For Extraversion, average and low category was score <121, high and very high ≥121. Openness domain was transformed into an average and low category with score <121, and high and very high category with ≥121. Agreeableness was divided into same categories with score under 137, and equal or above 137. Conscientiousness categories waere defines as score lower then 133, and equal or higher than 133.

The Zung Self-Rating Depression Scale (SDS) was used to assess depressive symptoms [29]. The SDS is a 20-item self-report questionnaire used as a screening tool. Each item is scored ranging from 1 to 4, and a total score is provided by summing item scores ranging from 25 to 100, with a higher score denoting greater depression. A depression state was indicated by the SDS score ≥ 50. Degree of depression was classified as mild depression with score 50–59, moderate with score 60–65 and severe depression with score ≥70.

Anxiety symptoms were assessed using the Zung Self-Rating Anxiety Scale (SAS) [30]. The SAS scale consists of 20 items, and each item is answered on a 4-point Likert-type scale ranging from “never” to “always”. Higher score means more serious anxiety symptoms. The presence of anxiety symptoms was defined as the SAS score ≥ 45. Degree of anxiety was classified as mild to moderate (40–59), severe (60–74) and most extreme severe (≥ 75).

### Statistical analyses

Continuous variables were expressed as the mean ± SD or median (range), whilst categorical data were expressed as n (%). A one way analysis of variance (ANOVA) with post-hoc analyses with Bonferroni correction were used to analyze the differences between groups in sociodemographic characteristics and personality of examined groups. Logistic regression analysis was performed in order to estimate the risk of personality abnormalities, depression and anxiety for pSS. Neuroticism, Extraversion, Openness to experience, Agreeableness and Conscientiousness were treated as dichotomous variables with average or lower as referent category and high and higher as the outcome. Classification of these domains was done according to McCrae RR [18]. The independent association of disease status with each personality profile and depression/anxiety status was subsequently analysed in separate logistic regression models, adjusted for potential confounders (age, education, type of settlement, marital status, community, satisfaction with family relationships, duration of the disease and comorbidity—diabetes mellitus, mild neurosis, hypothyroidism, and autoimmune hepatitis). The Five personality domains, depression and anxiety as dichotomous were dependent variables. Sample size of n = 211 respondents (3 groups), an error of the first type of 0.05 and study power (1-β) of 0.8 were sufficient to detect meaningful differences, with effect size f = 0.21. Also, the relative size of the effect was assessed based on standardized estimates of effect size according to Cohen’s benchmarks [31].

Statistical analyses were performed using SPSS [Version 16.0. SPSS Inc., Chicago, IL, USA]. *P* values less than 0.05 were considered significant.

## Results and discussion

### Clinical, sociodemographic characteristics and comorbidity in the study population

Among 105 pSS patients, none had high disease activity, 57 (64%) patients had moderate diasease activity (range 5–13) and 32 (36%) patients had low disease activity according to ESSDAI. The mean ESSPRI was 5.20. Xerophtalmia and xerostomia were present in 97/105 (92.4%) and 89/105 (84.8%) patients, respectively. Positive Schirmer test (≤5mm/5min) was found in 99/105 (95.1%) pSS patients and sialoscintigraphy in 74/87 (85.0%) pSS patients. Among pSS patients, 77% were anti-SSA/Ro antibody positive, 43% were anti-SSB/La antibody positive, 86% were ANA positive and 77% RF positive. Biopsy of LSG was positive in 60/82 (73.1%) pSS patients. There were no statistically significant differences in age between patients with pSS, RA and healthy controls (p = 1.00). The disease duration was longer in RA patients than in pSS patients (p = 0.001). Most patients had low educational level (p = 0.013), while the most of healthy controls were unemployed (p = 0.001). Refer to healthy controls, most patients live in urban settlement (p = 0.037). Cardiovascular morbidity (p = 0.001) and diabetes mellitus (p = 0.025) were the most diseases among RA patients in comparison with pSS patients and healthy controls. Neurosis and hypothyreosis were equally distributed in study groups (p = 0.559 and p = 0.242, respectively). The clinical, sociodemographic characteristics of study groups and comorbidity are presented in Table 1.

**Table 1:** Clinical, sociodemographic characteristics and comorbidity of the study groups.

	pSS patients (N = 105)	RA patients (N = 52)	Healthy Control (N = 54)	p Value
Clinical characteristics				
Age, mean (S.D.), years	51.34±10.25	51.37±10.79	51.35±9.75	1.000
Duration of disease, mean (S.D.), years	5.98±3.61	8.10±3.61	-	0.001**
ESSDAI (mean (S.D.); Median (range)	11.18±13.34 6.00 (0–75)	-	-	-
Low (0–4)	32 (36.0%)	-	-	-
Moderate (5–13)	57 (64.0%)	-	-	
High (≥14)	0	-	-	
ESSPRI (0–10),mean (S.D.) Median (range)	5.20±1.81 5.33(1.66–9.33)	-	-	-
DAS 28	-	4.01±1.16		
Socio-demographic characteristics				
Educational levelª, n (%)				
Low	78 (74.3)	38 (73.1)	26 (48.1)	0.013*
Medium	14 (13.3)	6 (11.5)	12 (22.2)	
High	13 (12.4)	8 (15.4)	16 (29.6)	
Work status, n (%)				
Employed	34 (32.4)	13 (25.0)	8 (14.8)	0.001**
Unemployed	42 (40.0)	20 (38.5)	39 (72.2)	
Retired	29 (27.6)	19 (36.5)	7 (13.0)	
Type of settlement, n (%)				
Urban	72 (68.6)	34 (65.4)	26 (48.1)	0.037*
Rural	33 (31.4)	18 (34.6)	28 (51.9)	
Marital status, n (%)				
With partner	75 (71.4)	33 (63.5)	35 (64.8)	0.522
Without partner	30 (28.6)	19 (36.5)	19 (35.2)	
Community, n (%)				
Living with someone	99 (94.3)	45 (88.5)	45 (85.2)	0.151
Living alone	6 (5.7)	6 (11.5)	8 (14.8)	
Satisfaction with familiar relations, n (%)				
Low	7 (6.7)	5 (9.6)	0 (0)	0.076
Medium	74 (70.5)	29 (55.8)	35 (64.8)	
High	24 (22.9)	18 (34.6)	19 (35.2)	
Comorbidity n (%)				
None	52 (49.5)	18 (34.6)	31 (57.4)	0.014*
One	32 (30.5)	18 (34.6)	20 (37.0)	
Two or more	21 (20.0)	16 (30.8)	3 (5.6)	
Type of disease				
Diabetes mellitus	10 (9.5)	9 (17.3)	1 (1.9)	0.025*
Neurosis	15 (14.3)	5 (9.6)	9 (16.7)	0.559
Hypothyreosis	23 (21.9)	9 (17.3)	6 (11.1)	0.242
Cardiovascular morbidity	31 (29.5)	27 (50.0)	10 (18.5)	0.001**

ESSDAI, EULAR Sjogren´s Syndrome Disease Activiy Index; ESSPRI, EULAR Sjogren´s Syndrome Patient Reported Index

ª Education: Low: primary school or lower vocational secondary education; Medium: intermediate general secondary education or intermediate vocational education; High: higher general secondary education or university education.

n %, number patients, percent

*P < 0.05 by ANOVA

**P < 0.01 by ANOVA.

### Personality characteristics, depression and anxiety in the study groups

Analysis of scores of each domains of personality between study groups with the effect size (d) between pSS patients and healthy controls are presented in Table 2. Patients with pSS had higher scores of Neuroticism (d = 0.46, p = 0.007) and lower scores of Extraversion (d = 0.51, p = 0.001) and Openness for experience (d = 0.65, p = 0.013) compared to healthy controls. Analysis of each subdomains of personality in study groups are presented in S1 Table. There was no significant differences between pSS group and healthy controls in the score of depression (d = 0.171, p>0.05). However, patients with pSS had significantly higher score of anxiety in comparison to healthy controls (p<0.0001). There were no differences between personality profiles patients with pSS and RA patients, independent for the duration of the disease. However, patients with RA shown tendency to more depression than pSS patients (p = 0.051).

**Table 2:** Domains of personality, depression and anxiety scores between study groups.

	pSS patients N = 105 (Mean, 95% CI)	RA patients N = 52 (Mean, 95% CI)	Healthy Controls N = 54 (Mean, 95% CI)	Effect size D	p*	p^a^
Neuroticism	94.76 (90.66–98.87)	95.56 (90.85–100.27)	84.63 (78.46–90.80)#	0.46	0.007	0.422
Extraversion	94.79 (91.55–98.03)	92.15 (87.87–96.44)	104.20 (98.67–109.73)#	0.51	0.001*	0.206
Openness to experience	101.86 (97.95–105.77)	102.69 (97.52–107.87)	111.17 (106.23–116.11)#	0.65	0.013*	0.858
Agreeableness	120.48 (118.06–122.89)	121.37 (117.13–125.60)	121.04 (117.65–124.42)	0.05	0.916	0.999
Conscientiousness	122.97 (119.77–126.17)	121.00 (116.29–125.71)	125.02 (120.30–129.74)	0.12	0.471	0.481
SDS	56.75 (55.55–57.94)	54.90 (53.22–56.58)	55.48 (53.76–57.21)	0.20	0.171	0.051
SAS	37.53 (35.50–39.56)	38.63 (35.93–41.33)	30.48 (28.20–32.77)#	0.74	<0.001	0.466

*according to one way ANOVA

#differences between means of Sjogren and healthy controls according to multiple comparisons Bonferroni test

d—Cohen`s d as the measure of effect size (pSS patients vs. Healthy controls)

p^a^according to ANCOVA between pSS and RA patients with disease duration as potential confound

Categories of personality domains, depression and anxiety patients in study groups are presented in Table 3. Patients with RA and pSS had more frequently high and very high degree of Neuroticism compared with healthy controls (p = 0.005). However, high and very high degree of Extraversion was statistically significant less frequent in pSS and RA patients compared to healthy controls (p<0.001). Degree of anxiety was higher in pSS and RA patients compared to healthy controls (p = 0.002), although the most patients had mild to moderate anxious (p = 0.005).

**Table 3:** Categories of personality domains, depression and anxiety patients in study groups.

Categories of personality domains, n (%)	Group			p*
	pSS N = 105	RA N = 52	Healthy controls N = 54	
Neuroticism, High and very high ≥96	54 (48.6)	27 (51.9)	14 (25.9)	0.005
Extraversion, High and very high ≥121	6 (5.7)	2 (3.8)	14 (25.9)	<0.001
Openness to experience, High and very high ≥121	14 (13.3)	10 (19.2)	15 (27.8)	0.084
Agreeableness, High and very high ≥137	14 (13.3)	7 (13.5)	4 (7.4)	0.504
Conscientiousness, High and very high ≥133	33 (31.4)	13 (25.0)	17 (31.5)	0.678
Depression, ≥50	20 (19.0)	6 (11.5)	6 (11.1)	0.294
Normal (25–49)	11 (10.5)	7 (13.5)	7 (13.0)	0.637
Mild depression (50–59)	64 (61.0)	37 (71.2)	31 (57.4)	
Moderate depression(60–69)	28 (26.7)	7 (13.5)	15 (27.8)	
Severe depression (≥70)	2 (1.9)	1 (1.9)	1 (1.9)	
Anxiety, ≥45	28 (26.7)	16 (30.8)	3 (5.6)	0.002
Normal (<45)	77 (73.3)	36 (69.2)	51 (94.4)	0.005
Mild to moderate anxiety (45–59)	25 (23.8)	16 (30.8)	3 (5.6)	
Severe anxiety (60–74)	3 (2.9)	0	0	
Most extreme anxiety (≥75)	0	0	0	

*according to chi-square test

### Personality characteristics, depression and anxiety in pSS patients and their association with sociodemographic factors and comorbidity

The results of logistic regression analyses of personality domains, depression and anxiety in pSS patients compared to healthy controls and RA patients are presented in Table 4. Patients with pSS had higher risk for Neuroticism (OR 3.025, p = 0.003), lower risk for Extraversion (OR 0.173, p = 0.01) and Openness to experience (OR 0.400, p = 0.028) and also lower risk for anxiety compared to healthy controls (OR 6.182, p = 0.004).

**Table 4:** Odds ratios for personality domains, depression and anxiety in pSS patients, healthy controls and patients with RA.

Personality domains	pSS vs Healthy			pSS vs RA			pSS vs. RA^a^		
	OR	95% CI	p*	OR	95% CI	p*	OR	95% CI	p*
Neuroticism	3.025	1.47–6.21	0.003	0.98	0.50–1.91	0.953	0.796	0.39–1.61	0.527
Extraversion	0.173	0.06–0.48	0.001	1.51	0.29–7.78	0.619	1.684	0.32–8.84	0.538
Openness to experience	0.400	0.18–0.91	0.028	0.646	0.26–1.57	0.336	0.826	0.32–2.14	0.694
Agreeableness	1.923	0.60–6.16	0.271	0.989	0.37–2.62	0.982	0.877	0.32–2.39	0.798
Conscientiousness	0.998	0.49–2.02	0.995	1.375	0.65–2.91	0.406	1.442	0.66–3.15	0.359
Depression	1.882	0.71–5.00	0.205	1.804	0.68–4.80	0.238	1.623	0.60–4.41	0.343
Anxiety	6.182	1.78–21.41	0.004	0.818	0.39–1.70	0.590	0.834	0.39–1.79	0.641

*according to logistic regression analysis

^a^Logistic regression models adjusted for the duration of the disease

In the multivariate model adjusted for potential confounder (Table 5), pSS patients were more likely to show higher degree of Neuroticism (p = 0.010), lower degree of Extraversion (p = 0.008) and higher level of anxiety (p = 0.004) in comparison with healthy controls. Psychological profiles of patients with RA were comparable to patients with pSS. In models adjusted for all potential confounders, education level was associated with Openness to experience (p = 0.002). Younger patients show tendency toward higher Openness to new experience, but difference was not statistically significant (p = 0.056). Satisfaction with family relationships (p = 0.011) was significantly associated with high degree of Extraversion and Conscientiousness (p = 0.011 and p = 0.033, respectively). Neither of potential confounders (age, education, type settlement, marital status, community, satisfaction with familiy relationships, duration of the disease and comorbidity) were associated with the level of anxiety.

**Table 5:** Associations of personality domains, depression and anxiety in pSS patients and healthy controls by multivariate logistic regression analysis.

Variables	OR (95% CI) P						
	Domains of personality					Self-rating Depression Scale	Self-rating Anxiety Scale
	Neuroticism	Extraversion	Openness to experience	Agreeableness	Conscientiousness		
RA patients	1.004 (0.50–20.3) 0.991	0.543 (0.09–3.43) 0.516	1.626 (0.58–4.59) 0.359	1.106 (0.39–3.12) 0.849	0.612 (0.27–1.37) 0.232	0.533 (0.19–1.46) 0.222	1.085 (0.51–2.30) 0.832
Healthy controls	0.367 (0.17–0.79) 0.010	4.854 (1.51–15.62) 0.008	1.797 (0.70–4.62) 0.224	0.440 (0.12–1.55) 0.201	0.809 (0.38–1.74) 0.586	0.541 (0.19–1.51) 0.242	0.156 (0.04–0.56) 0.004
pSS patients	Reference	Reference	Reference	Reference	Reference	Reference	Reference
Confounders							
Age	1.022 (0.99–1.05) 0.170	0.961 (0.92–1.01) 0.113	0.964 (0.93–1.00) 0.056	1.041 (0.99–1.09) 0.107	1.009 (0.98–1.04) 0.588	0.979 (0.94–1.02) 0.288	0.989 (0.95–1.02) 0.547
Education	0.812 (0.52–1.25) 0.348	1.329 (0.68–2.58) 0.402	2.222 (1.33–3.71) 0.002	1.170 (0.63–2.17) 0.620	1.466 (0.94–2.27) 0.089	0.958 (0.55–1.68) 0.880	1.000 (0.59–1.69) 0.992
Type of settlement	0.804 (0.54–1.20) 0.291	1.025 (0.50–2.11) 0.947	1.506 0.410 (0.85–2.67) 0.162	0.907 (0.49–1.68) 0.758	0.998 (0.64–1.54) 0.992	1.315 (0.75–2.30) 0.338	0.860–0.151 (0.53–1.40) 0.547
Marital status	1.284 (0.94–1.75) 0.118	0.697 (0.36–1.36) 0.291	0.783 (0.49–1.24) 0.299	0.999 (0.64–1.56) 0.996	0.854 (0.60–1.21) 0.375	0.888 (0.57–1.38) 0.600	1.290 (0.91–1.83) 0.155
Community	1.055 (0.88–1.26) 0.558	1.041 (0.74–1.46) 0.816	1.208 (0.94–1.55) 0.137	1.278 (0.99–1.65) 0.061	1.009 (0.83–1.22) 0.925	1.025 (0.81–1.30) 0.840	0.928 (0.75–1.15) 0.928
Satisfaction with familiar relationship	0.726 (0.42–1.26) 0.258	4.144 (1.38–12.43) 0.011	1.342 (0.64–2.83) 0.440	1.445 (0.62–3.37) 0.394	1.971 (1.06–3.68) 0.033	1.255 (0.60–2.64) 0.548	1.039 (0.54–1.98) 0.908
Comorbidity	1.103 (0.73–1.66) 0.641	0.326 (0.10–1.01) 0.326	0.868 (0.46–1.63) 0.660	0.776 (0.41–1.46) 0.433	1.302 (0.83–2.03) 0.245	1.223 (0.71–2.12) 0.473	1.256 (0.78–2.02) 0.349
Adjusted R Square, P	0.14; 0.099	0.38; <0.001	0.28; <0.001	0.08; 0.409	0.08; 0.162	0.04; 0.792	0.12; 0.039

## Discussion

Chronic diseases influence personality development, and common chronic conditions such as heart disease, stroke, diabetes, cancer, hypertension, arthritis and respiratory disease were found to consistently decrease the level of Extraversion, Conscientiousness and Openness to experience and increase the level Neuroticism in affected patients [32]. These findings are of clinical importance as patients’ personality characteristics may be associated with adherence and response to pharmacological treatment [33, 34].

The current study aimed to identify psychological profiles and personality traits among patients suffering from pSS. In this study, we have tried to gain more insight into the ways in which patients’ personality patterns interact with styles of coping with chronic illness. We recruited patients with a diagnosis of pSS, according to current diagnostic criteria. We have chosen to compare this group of patients with healthy age- and gender-matched healthy controls and with a group of patients suffering from another chronic inflammatory rheumatic disease such as RA.

To our best knowledge, this study is the first attempt to assess personality dimensions in pSS patients using the Five-Factor Model (FFM). Our findings demonstrate that patients with pSS have higher Neuroticism, lower Extraversion and lower Openness to experience in comparison to healthy subjects. Our results also show that pSS patients have specific personality traits that are similar to RA patients. Among rheumatic diseases, the assessment of personality dimensions by FFM has been used only in patients with fibromyalgia in whom Neuroticism, Openness to experience and Agreeableness were associated with severity of pain, sleep disturbances, fatigue and confusion [35, 36]. Recent study by Bucourt E et al. [37] revealed that high Neuroticism and low Conscientiousness (high impulsivity) were associated with high level of chronic pain, which is of importance as fibromyalgia may be one of the clinical manifestations of pSS.

The higher scores of Neuroticism in pSS patients in comparison to healthy subjects are in line with the similar findings in other chronic diseases [32, 38, 39]. We confirmed that patients with pSS have higher risk for greater Neuroticism compared to healthy controls. Neuroticism or Emotional lability is „deconstructing”personality trait in individuals who are unable to cope with stress effectively. Higher levels of Neuroticism found in patients with pSS and RA in this study point to emotional instability and difficulties in social adaptation of patients with chronic rheumatic diseases. In general, these patients tend to experience negative emotions and are therefore more vulnerable, less capable of exerting control over impulsive behavior and stress. Being highly emotional they constantly worry and overreact to external stimuli which could affect their physical functions, quality of life and overall treatment outcome. Neither of sociodemographic factors were associated with the high level of Neuroticism in our group.

Extraversion is the most relevant predictor of subjective well-being (SWB) [40]. Extraversion is related to positive affect and more positive subjective evaluations of daily activities. In this context, high level of Extraversion is a protective factor against the effects of stress. In our study, patients had low level of Extraversion, making them vulnerable to stress. Low Extraversion can be explained by lower prevalence of high patients᾽ satisfaction with family relationships. This suggests that family support influence to more favorable life events in patients with pSS.

High level of Neuroticism and low level of Extraversion in patients with pSS in our study are consistent with the stress-vulnerability model in autoimmune diseases. Low distress tolerance may contribute to the development of autoimmune disorders. Stress reactions or persistent stress activate the hypotalamic-pituitary-adrenal (HPA) axis and the sympathetic-adrenal-medullary (SAM) system which may have proinflammatory effects in autoimmune diseases [41]. Eysenk et al. [42] have shown that high level of Neuroticism is associated with higher activity of certain parts of sympathetic autonomic nervous system which resulted in overreaction to external stimuli with prolonged duration. Savic et al. [43] investigated the association between hypothalamo-pituitary adrenal (HPA) axis and memory and showed that the basal cortisol level is a biologic marker for stress. Moreover, neuroticism has been linked to reduced cell-mediated immunity, increased levels of pro-inflammatory cytokines and lower cortisol stress reactivity [44]. Persons experiencing chronic stressful life events can become stronger or more vulnerable depending on their ability to cope with stress. High level of Neuroticism is related to poor capacity to cope with stress and may lead to negative self-esteem and depression. High level of Neuroticism has been associated with increased risk for development of depressive and anxiety disorders [45, 46], which is in accordance with higher prevalence of anxiety in pSS patients observed in this study. Also, patients with RA showed tendency to more depression than patients with pSS.

In our study, low Openness to experience can be explained by lower education, which is line with the studies that demonstrated lifetime protective effect of education against distress [47,48]. Higher patients᾽ education may contribute to their better understanding of the disease and positively affect the disease outcome. In our study, younger patients showed tendency to more opened to new experience, while older patients were less imaginative and creative. This is similar to decreased Extraversion, Conscientiousness and Openness in older age in other study [49,50].

Levels of Agreeableness and Conscientiousness were similar between pSS patients and healthy subjects and good satisfaction with family relationships predicted high level of Conscientiousness in pSS patients. Agreeableness has protective effect against social stressors and is related to happiness and extraversion [51]. Conscientiousness, as opposed to neuroticism, predicts lower reactivity to daily stressors and lower average level of negative affect [52, 53]. In our study, satisfaction with familiar relationship was associated with higher level of Conscientiousness.

Although FFM is widely accepted and universal method for describing personality traits it has potential limitations. For example, Neuroticism is clinically heterogeneous and diagnostically nonspecific and could be present in neurotic disorders, but also in psychotic and affective disorders or in healthy persons as demonstrated in study by Ben-Porath and Waller [54]. From a clinical point of view it is surprising that traits such as anxiety, depression and impulsiveness are present in the same dimension of Neuroticism. Watson et al. [55] reported correlation between anxiety and Neuroticism and negative correlation between depression and positive emotionality (E) and impulsiveness and with low level of control (C), the data that cast doubt on the reliability and the validity of Neuroticism scale (N).

Prevalence of depression and anxiety in patients with pSS in this study is comparable to recently published results of Milin et al. [46]. They found that anxiety is more common than depression in pSS patients which should be taken into account when managing all patients with sicca symptoms. In our study, the most of pSS patients had mild to moderate degree of anxiety. Only two patients with pSS had severe depression. Patients with pSS experienced similar levels of depression as healthy controls and the two-thirds of patients had mild depression. The possible explanation of rather high depressive symptoms in healthy subjects is that people in Serbia experienced many social and economic difficulties in recent years that may have brought depression and hopelessness. The general situation in the country, the recent economic crisis, and the consequences of war in the surrounding countries and in Serbia could have an impact on mental health in general population [56].

The limitations in this study concern the fact that all subjects included in the study were females so that obtained data may not be applicable to men with pSS. Additionally, our groups were not comparable refer to educational level, work status and type of settlement. This is a big limitation our study.

In summary, we have demonstrated that there are associations between environmental factors and psychological personality dimensions. Although, the pSS subjects have a greater comorbidity, no association is observed between comorbidity and psychological profile. Our results suggested that comorbidity does not affect psychological profile of patients.

## Conclusions

We confirmed that pSS patients have psychological profiles and levels of anxiety different to healthy subjects. In our study, patients with pSS were emotionally unstable, introverted, and more anxious than healthy controls. Education and satisfaction with family relationships were significant predictors for psychological characteristics of patients, independently of clinical diagnosis. The better understanding of personality dimensions in patients with pSS may provide adequate help by professionals in overcoming adaptation problems which have been observed in these patients.

## Supporting information

S1 Table Domains and subdomains of personality, depression and anxiety scores between study groups. (DOCX)
