# Supplementary material for: Personality, depression and anxiety in primary Sjogren's syndrome – Association with sociodemographic factors and comorbidity
Source: PLoS One. 2019 Jan 17;14(1):e0210466. doi: 10.1371/journal.pone.0210466 (PMC6336324; doi:10.1371/journal.pone.0210466)
Supplement: S1 Table — (DOCX) [file pone.0210466.s001.docx]

|  | pSS patients  N=105  (Mean, 95% CI) | RA patients  N=52  (Mean, 95% CI) | Healthy Controls  N=54  (Mean, 95% CI) | Effect size  d | p^*^ | p^a^ |
| --- | --- | --- | --- | --- | --- | --- |
| Neuroticism | 94.76 (90.66 – 98.87) | 95.56 (90.85 - 100.27) | 84.63 (78.46 - 90.80)# | 0.46 | 0.007 | 0.422 |
| Anxiety | 17.93 (16.89 – 18.98) | 17.38 (16.21 – 18.55) | 16.22 (14.16 – 18.29) | 0.26 | 0.213 | 0.954 |
| Angry Hostility | 15.77 (15.03 – 16.51) | 16.02 (14.99 – 17.05) | 13.39 (12.35 – 14.42)**#** | 0.63 | <0.001 | 0.199 |
| Depression | 14.94 (13.95 – 15.93) | 14.69 (13.37 – 16.02) | 12.22 (10.79 – 13.65)**#** | 0.52 | 0.005 | 0.789 |
| Self-consciousness | 16.25 (15.40 – 17.09) | 17.35 (16.24 – 18.45) | 14.46 (13.10 – 15.82) | 0.38 | 0.004 | 0.172 |
| Impulsivenes | 16.18 (15.46 – 16.90) | 16.15 (15.12 – 17.19) | 16.83 (15.75 – 17.91) | 0.17 | 0.540 | 0.818 |
| Vulnerability | 13.69 (12.80 – 14.57) | 13.96 (12.69 – 15.23) | 11.94 (10.57 – 13.32) | 0.36 | 0.047 | 0.459 |
| Extraversion | 94.79 (91.55 – 98.03) | 92.15 (87.87 – 96.44) | 104.20 (98.67 – 109.73)# | 0.51 | 0.001* | 0.206 |
| Warmth Mean | 20.06 (19.29 – 20.83) | 19.06 (18.08 – 20.04) | 21.11 (19.95 – 22.27)**#** | 0.25 | 0.030 | 0.071 |
| Gregariousness | 16.17 (15.26 – 17.08) | 15.94 (14.61 - 17.27) | 18.43 (17.11 to 19.75)**#** | 0.47 | 0.009 | 0.578 |
| Assertivenes | 11.91 (11.17 – 12.66) | 11.52 (10.63 – 12.41) | 14.19 (13.15 – 15.22) | 0.60 | <0.001 | 0.441 |
| Activity | 17.59 (16.75 – 18.43) | 15.81 (14.70 – 16.92) | 19.00 (16.98 – 21.02) | 2.87 | 0.008 | 0.024 |
| Excitement-Seeking | 12.25 (11.45 – 13.05) | 12.85 (11.69 – 14.00) | 12.98 (11.83 – 14.13) | 0.25 | 0.500 | 0.647 |
| Positive Emotions | 16.81 (15.90 – 17.72) | 16.98 (15.71 – 18.25) | 18.50 (17.10 – 19.90) | 3.04 | 0.097 | 0.920 |
| Openess to experience | 101.86 (97.95 – 105.77) | 102.69 (97.52 – 107.87) | 111.17 (106.23 – 116.11)# | 0.65 | 0.013* | 0.858 |
| Fantasy | 14.79 (13.72 – 15.86) | 14.62 (12.90 – 16.33) | 16.33 (14.88 – 17.79) | 0.28 | 0.198 | 0.618 |
| Aesthetics | 19.53 (18.48 – 20.58) | 19.92 (18.67 – 21.18) | 21.09 (19.69 – 22.57) | 0.29 | 0.203 | 0.778 |
| Feelings | 19.37 (18.48 – 20.26) | 20.10 (18.96 – 21.23) | 20.85 (19.64 – 22.07) | 0.33 | 0.133 | 0.366 |
| Actions | 14.51 (13.81 – 15.22) | 14.06 (13.12 – 14.99) | 14.56 (13.62 – 15.49) | 0.01 | 0.702 | 0.299 |
| Ideas | 15.07 (14.02 – 16.11) | 15.88 (14.60 – 17.17) | 18.19 (16.67 – 19.70)**#** | 0.57 | 0.002 | 0.664 |
| Values | 18.58 (17.92 – 19.24) | 18.12 (17.11 – 19.12) | 20.15 (19.18 – 21.12)**#** | 0.45 | 0.006 | 0.178 |
| Agreeableness | 120.48 (118.06 – 122.89) | 121.37 (117.13 – 125.60) | 121.04 (117.65 – 124.42) | 0.05 | 0.916 | 0.999 |
| Trust | 18.60 (17.79 – 19.41) | 18.96 (17.79 - -20.14) | 20.13 (18.98 – 21.28) | 0.52 | 0.093 | 0.768 |
| Straightforwardness | 21.99 (21.16 – 22.83) | 21.87 (20.74 – 22.99) | 21.94 (20.86 – 23.03) | 0.01 | 0.984 | 0.216 |
| Altruism | 21.66 (21.08 – 22.23) | 21.79 (20.99 – 22.59) | 22.26 (21.31 – 23.21) | 0.18 | 0.503 | 0.876 |
| Compliance | 18.66 (17.82 – 19.50) | 18.50 (17.26 – 19.74) | 18.35 (17.25 – 19.45) | 0.07 | 0.911 | 0.855 |
| Modestly | 18.79 (18.07 – 19.51) | 19.48 (18.30 – 20.67) | 17.28 (16.08 – 18.48) | 0.37 | 0.016 | 0.471 |
| Tender-Mindedness | 20.78 (20.22 – 21.34) | 20.77 (19.84 – 21.70) | 21.07 (20.06 – 22.09) | 0.09 | 0.844 | 0.949 |
| Conscientiousness | 122.97 (119.77 – 126.17) | 121.00 (116.29 – 125.71) | 125.02 (120.30 – 129.74) | 0.12 | 0.471 | 0.481 |
| Competent | 21.17 (20.47 – 21.87) | 21.13 (20.08 – 22.19) | 22.02 (20.80 – 23.24) | 0.21 | 0.376 | 0.608 |
| Order | 19.16 (18.47 – 19.85) | 18.44 (16.69 – 20.19) | 19.44 (18.31 – 20.58) | 0.07 | 0.494 | 0.376 |
| Dutifulness | 22.16 (21.52 – 22.81) | 21.90 (21.08 – 22.73) | 21.59 (20.79 – 22.40) | 0.18 | 0.554 | 0.829 |
| Achievement Striving | 20.32 (19.49 – 21.16) | 19.75 (18.46 – 21.04) | 21.26 (20.08 – 22.44) | 0.22 | 0.200 | 0.549 |
| Self-Discipline | 20.93 (20.13 – 21.73) | 20.33 (19.09 – 21.57) | 21.26 (19.88 – 22.63) | 0.07 | 0.549 | 0.409 |
| Deliberation | 19.22 (18.36 – 20.08) | 19.44 (18.41 – 20.47) | 19.44 (18.07 – 20.82) | 0.05 | 0.935 | 0.800 |

^*^according to oneway ANOVA

#differences between means of Sjogren and healthy controls according to multiple comparison Bonferroni test

d - Cohen`s d as the measure of effect size (pSS patients vs. Healthy controls)

p^a^according to ANCOVA between pSS and RA patients with disease duration as potential confound
